# Interleukin-10 Producing Regulatory B Cells Transformed CD4^+^CD25^−^ Into Tregs and Enhanced Regulatory T Cells Function in Human Leprosy

**DOI:** 10.3389/fimmu.2018.01636

**Published:** 2018-07-23

**Authors:** Mohd. Tarique, Huma Naz, Santosh V. Kurra, Chaman Saini, Raza Ali Naqvi, Reeta Rai, Mohd Suhail, Neena Khanna, Donthamshetty N. Rao, Alpana Sharma

**Affiliations:** ^1^Department of Biochemistry, All India Institute of Medical Sciences (AIIMS), New Delhi, India; ^2^Centre for Interdisciplinary Research in Basic Sciences, Jamia Millia Islamia, New Delhi, India; ^3^King Fahd Medical Research Center, King Abdulaziz University, Jeddah, Saudi Arabia; ^4^Department of Dermatovenerology, All India Institute of Medical Sciences (AIIMS), New Delhi, India

**Keywords:** leprosy, regulatory B cells, Tregs, interleukin-10, FoxP3, Teff cells

## Abstract

Regulatory B cells (Bregs) are known to exhibit their regulatory functions through interleukin-10 (IL-10) cytokine which suppress inflammation. There are only a few studies explaining the phenotype and functioning of these cells in contribution to host immunity in leprosy. Here, we evaluated the role of IL-10 producing Bregs in the pathogenesis of leprosy and assessed their immunoregulatory effects on Tregs and effector T cells. We found an increased frequency of Bregs and increased expression of their immune modulatory molecules (IL-10, FoxP3, and PDL-1) in leprosy patients. The potential immunoregulatory mechanism of Bregs was also investigated using MACS sorted Teff (CD4^+^CD25^−^) and Treg (CD4^+^CD25^+^) cells were cocultured with Bregs to elucidate the effects of Bregs on effector T and regulatory T cells. Cell coculture results showed that purified Bregs cells from leprosy patients convert CD4^+^CD25^−^ cells into CD4^+^CD25^+^ cells. Cell coculture experiments also demonstrated that leprosy derived IL-10 producing Bregs enhance FoxP3 and PD-1 expression in Tregs and enhanced Tregs activity. Blocking of IL-10 receptor confirmed that IL-10 producing Breg has immunomodulatory effect on Tregs and effector T cells as effector T cells are not converted into Tregs and enhanced expression of FoxP3 and PD-1 was not observed on Tregs. Collectively, these findings demonstrate that IL-10 producing Breg cells play an important mechanism in controlling the immunopathogenesis of leprosy and have an immunomodulatory effect on Tregs and effector T cells. Our findings may pave way for novel targets of IL-10 producing Bregs for immunotherapy in leprosy patients.

## Introduction

Leprosy is a chronic bacterial disease caused by *Mycobacterium leprae*, which primarily affects macrophages and Schwann cells from the peripheral nerves. It provides an ideal model to study the host–pathogen interaction and immunological dysregulation in humans as each clinical manifestation is associated with different levels of immune response to the *M. leprae* (1). Leprosy is classified into five clinical forms, tuberculoid (paucibacillary, BT/TT) pole which is characterized by the Th1 immune response, high cell-mediated immunity, relative resistance to the pathogen, and localized infection. While, lepromatous (multibacillary, BL/LL) pole the infection is associated with Th2 immune response, defective cell-mediated immune response, foamy macrophages in the dermis due to a very high number of bacilli, lesion on all over the body parts (2, 3). Three immunologically unstable form lies in-between these forms, borderline tuberculoid (BT), borderline-borderline, and borderline lepromatous leprosy, presenting wavering characteristics between the two poles of the disease. Previously, our laboratory had observed Th3 type immune response with the progression of leprosy (tuberculoid to lepromatous leprosy) (4). Furthermore, we also observed an increased frequency of IL-35-producing Tregs in BL/LL pole of leprosy (5) and also changed in the plasticity of Tregs upon IL-12 and IL-23 treatment (6). Recently, we also reported that another immunosuppressive population γδ T cells increased in the leprosy patients (7) and defective T cell immune response in leprosy (8, 9).

Traditionally, B cells have been thought to as antigen-presenting cell (APC) and antibody producing cell (10). It is one of the least studied immune cell in leprosy. Recent studies have shown that the role of B cells extends beyond the production of antibodies and APC, the negative regulative effect of B cell by producing regulatory cytokine have been identified and termed “regulatory B cells” (10). A variety of regulatory B cell (Breg) subsets have been identified, interleukin-10 (IL-10)-producing Bregs in a murine model of experimental autoimmune encephalomyelitis (EAE) (11), in humans (12) and TGF-β1 producing B cells when stimulated with LPS *in vitro* (13). Among these subsets, IL-10 producing B cell (B10) is the most widely studied Breg subset. The most prominent effector function of Bregs is the production of the potent immunosuppressive cytokine IL-10 which is the hallmark cytokine of Bregs. Bregs have ability to modulate the immune responses by acting on different cell types, such as dendritic cells (DC) (14), macrophages (15) as well as suppress inflammation by restoring the balance between Th1/Th2 (16, 17), regulates CD4^+^ T cell activation *in vitro* (18), inhibiting the antigen presenting cells activity, suppresses inflammatory cytokine production by T cells, and induces apoptosis in target effector cells (19). In this study, we aim to elucidate the effect of IL-10 producing Bregs derived from leprosy patients on effector T cells and Tregs activity.

Several studies showed that Tregs upregulated in the leprosy patients and resulted in the suppression of the host immune responses (8, 20). Numerous mechanisms may bestow the dysfunction of *M leprae* specific T cells, such as enrichment of pathogen and, suppressive cytokines IL-10 and TGF-β secreted by Tregs and T cells. These changes eventually lead to gradual loss of T-cell function and cause *M leprae* specific T cells anergy. IL-10 polymorphism has also seen in the North Indian population also associated with fast progression of the disease (21). In the immunosuppressive environment like leprosy, upregulation of inhibitory molecules such as cytotoxic T-lymphocyte-associated antigen-4 (CTLA-4) and programmed cell death-1 (PD-1) on T cells and their ligands on APCs which resemble T-cell anergy and exhaustion in leprosy patients (4, 22). PD-1 which is an inhibitory costimulatory molecule employs its effect on T cells by interfering the function and downregulating the cytokine production (IFN-γ, TNF-α, and IL-2) and cell proliferation (23). The PD-1-PD-L pathway plays one of the crucial role in dampening the T cell immune responses during many infectious diseases.

In this maiden attempt to study, we elucidated the role of B regulatory cells in leprosy which never explored before. Our results show an increase in IL-10^+^ Bregs and PDL-1 expression in *in vitro* antigen induced peripheral blood mononuclear cell (PBMCs) of leprosy patients. We also observed the change in the phenotype of Teff and Tregs under the influence of IL-10 producing Bregs and enhanced regulatory T-cells function in human leprosy.

## Materials and Methods

### Patients and Controls

In this study, we recruited 50 newly diagnosed leprosy patients, 25 BT, and 25 Lepromatous leprosy (BL/LL) from Department of Dermatology, All India Institute of Medical Sciences (AIIMS) New Delhi, India. Patients below 18 years of age, tuberculosis, pregnant women, HIV, diseased, and MDT treatment were not included in this study. Leprosy patients were determined by clinical and histological criteria on the basis of Ridley–Jopling classification. In the addition age match, 10 healthy volunteers were recruited after receiving the written consent (Table 1).

**Table 1:** Clinical details of 50 newly diagnosed untreated leprosy patients and 10 HC subjects.

Clinical types	Number of Patients	Sex		Age (years)	BI	Duration of Disease (years)
					
		M	F			
BT	25	14	11	19–56	0–0.4	0.7–2.0
BL/LL	25	15	10	21–57	4.8–6	0.8–1.9
HCs	10	05	05	22–53	–	–

*Patients were typed based on Ridley–Jopling classification, BI (mean of six lesional sites), and skin lesions*.

*M, male; F, female; BT, borderline tuberculoid; BL, borderline lepromatous; LL, lepromatous leprosy; HC, healthy controls; BI, bacillary index*.

### Ethics

Ethical approval was obtained from the Institute Ethics Committee, AIIMS, New Delhi, India (IESC/T-417/01.11.2013). Written consent was informed obtained from the patients prior to take blood sample.

### PBMCs Isolation and *In Vitro* Culture

Blood samples were layered on ficoll-hypaque (Sigma Aldrich, USA), and mononuclear cells were isolated by centrifugation at 1,500 rpm for 25 min. Cells were washed thrice in sterile PBS by centrifugation at 1,500 rpm for 10 min. Washed cells were resuspended in RPMI 1640 along with 10% fetal calf serum (Gibco, CA, USA) and cell viability and enumeration were estimated by 0.2% trypan blue using hemocytometer. 1 × 10^6^ cells/ml were stimulated with *M. leprae* sonicated antigen (10 µg/ml) kindly provided by P. J. Brennan of Colorado State University. All the cultures were stimulated with IL-2, anti-CD3/CD28. After stimulation, cultures were incubated in 5% CO_2_ incubator at 37°C for 48 h. After harvesting the cultured cells, they were processed for FACS staining.

### Cell Coculture

CD19^+^ B cells were immediately isolated from all three groups [Healthy controls (HCs), tuberculoid (BT) and lepromatous (BL/LL)] after the blood was drawn from the patients by using magnetic-activated cell sorting (MACS) technology (Miltenyi Biotech, Auburn, CA, USA). CD4^+^CD25^+^ Tregs and CD4^+^CD25^−^ T effector was separately isolated by positive selection and negative selection according to a magnetic column based system Tregs/Teff cell isolation kit (Miltenyi Biotech, Auburn, CA, USA) from healthy controls. Purity of MACS sorted CD19^+^, CD4^+^CD25^−^ T and CD4^+^CD25^+^ T cells were checked by flow cytometry. CD19^+^ (1 × 10^5^) cells from all three groups were cocultured with either CD4^+^CD25^−^ T cells (1 × 10^6^) or CD4^+^CD25^+^ T cells (1 × 10^5^). Coculture experiments were performed in Transwell system (Corning) in RPMI medium with 10% FBS. After seeding 1 × 10^5^ CD19^+^ cells in the bottom well, 1 × 10^6^ CD4^+^CD25^−^ T cells or 1 × 10^5^ CD4^+^CD25^+^ T cells were seeded on the upper mesh (pore size: 0.4 µm). Cells were collected for analysis of flow cytometry and qPCR after 48 h of the coculture. In the blocking experiment, either CD4^+^CD25^−^ T cells (1 × 10^6^) or CD4^+^CD25^+^ T cells (1 × 10^5^) were seeded in the bottom and blocked with purified antibodies against IL-10 receptor and 1 × 10^5^ CD19^+^ cells were seeded in the upper mesh. Isotype controls used as negative control and TGF-β (20 ng/ml) were used as positive control in coculture experiments. After 48 h, CD4^+^CD25^−^ T cells and CD4^+^CD25^+^ T cells were processed for FACS and qPCR.

### Flow Cytometer Staining

After 48 h, cultured cells were harvested and stained with surface antibodies Alexa Fluor 488 Mouse Anti-Human CD4 (RPA-T4), APC Mouse Anti-Human CD19 (Clone: HIB19), APC-Cy7 Mouse Anti-Human CD25 (Clone: M-A251), PE-Cyanine7 Anti-Human CD274 [programmed death-ligand 1 (PD-L1), B7-H1] (Clone: MIH1) and for 60 min at 4°C in the dark. After surface staining, cells were incubated with FoxP3 staining buffer for 15 min at room temperature; cells were washed twice and permeabilized with 1× permeabilization buffer (eBioscience) 30 min at room temperature. The cells were washed twice, resuspended in staining buffer, and incubated with PE Rat Anti-Human IL-10 (Clone: JES3-19F1) and PerCP-Cy5.5 Mouse Anti-Human FoxP3 (Clone: 236A/E7). Staining was performed according to the specifications of the manufacturer. All the antibodies were obtained from BD Biosciences, San Diego, CA, USA. The cells were fixed in 400 µl of 2% paraformaldehyde and stored at 4°C. For intracellular staining cultures were incubated with Protein Transport Inhibitor containing Monensin (BD Golgi Stop) for 4 h prior to harvest to block secretion of cytokine. The data were collected using FACS Canto flow cytometer (BD Biosciences) and analyzed cytometry along with isotype controls of phycoerythrin (PE mouse IgG1), Alexa Fluor 488 (mouse IgG1), APC Cy7 (mouse IgG1), APC (mouse IgG1) using FlowJo software.

### RNA Extraction and Real-Time PCR

Total RNA was extracted from cocultured CD4^+^CD25^+^ Tregs and CD4^+^CD25^−^ T effector cells using TRIzol reagent (Invitrogen, Carlsbad, CA, USA), according to the manufacturer’s protocol. The RNA quality and concentration were determined using UV spectrophotometer. Primers were designed by using www.ncbi.nlm.nih.gov/gene (Table 2). For cDNA synthesis, 1 µg total RNA was transcribed with cDNA Synthesis Kits (Thermo Scientific, USA). Reactions were performed according to the manufacturer’s instructions and the cDNA stored at −20°C until further use. Triplicate samples of cDNA from cocultured from each group was amplified in 384 well plates containing primers for the genes of interest, cytokines IL-10, PD-1, CTLA-4, FoxP3, and TGF-β and housekeeping gene GAPDH in Roche, LightCycler^®^ 480 System. Data were analyzed as threshold cycle values were normalized and expressed as DCt: mean Ct of gene of interest − mean Ct.

**Table 2:** Real-time PCR primers used in this study.

Genes	Gene ID	Sequence	Product length
*GAPDH*	*NM_001256799.2*	Forward 5′-GAAATGAATGGGCAGCCGTT-3′ Reverse 5′-A CGCCCAATACGACCAAATCA-3′	173
*FoxP3*	*XM_011543916.2*	Forward 5′-AGAGTTCCTCCACAACATGG-3′ Reverse 5′-CTCCCCAATGTGCCTATGAG-3′	280
*PD-1*	*NM_005018.2*	Forward 5′-CTCAGGGTGACAGAGAGAAG-3′ Reverse 5′-TGAGGGGTCCTCCTTCAG-3′	219
*CTLA-4*	*NM_001037631.2*	Forward 5′-AGGGAAGTTTTGTGGAGGAG-3′ Reverse 5′-TCAACTCAGATACCACCAGC-3′	209
*TGF-*β	*NM_000660.6*	Forward 5′-TGTCCAACATGATCGTGCG-3′ Reverse 5′-CAATGACACAGAGATCCGC-3′	197
*IL-10*	*NM_000572.2*	Forward 5′-CTGATACCTCAACCCCCATT-3′ Reverse 5′-CAGGGAAAACAGCTCAACAG-3′	267

### ELISA

Interleukin-10 cytokine was estimated by ELISA (Ready Set Go, eBioscience, San Diego, CA, USA) as per manufacturer’s instructions. Serum was tested in duplicate in 96-well plates (Nunc, Rochester, NY, USA) precoated with biotin conjugated anti-human antibodies IL-10. Protocol was followed according to the manufacturer’s instructions. The optical density of each well was read at 450 nm.

### Statistical Analysis

Differences among groups were evaluated using two tailed Mann–Whitney nonparametric and Student’s *t*-test for unpaired samples were performed using Graph Pad Prism version 5 (Graph Pad Software, Inc., San Diego, CA, USA). *p* < 0.05 was considered as statistically significant.

## Results

### Increased CD19^+^IL-10^+^ Cells in BT and BL/LL Leprosy Patients

**Figure 1:**
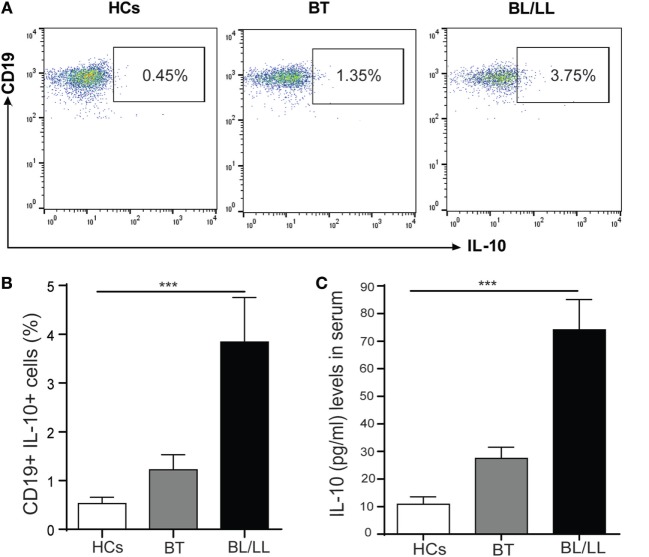
B regulatory cells in Leprosy. **(A)** Representative FACS plot showing enumeration of regulatory B cell (CD19^+^IL-10^+^) frequency in peripheral blood mononuclear cells isolated from peripheral blood of HCs, borderline tuberculoid (BT), and BL/LL patient cultured with *M. leprae* sonicated antigen for 48 h. **(B)** Graph Plots are showing total interleukin-10 (IL-10) producing CD19^+^ cells in BT and BL/LL (*n* = 25) vs. Healthy Contacts (*n* = 10). **(C)** Graph plot is showing IL-10 level in the serum of HCs and leprosy patients (BT, BL/LL). Mean ± SD values are shown in each set while *p* value <0.05 was considered significant. 100,000 cells were acquired and analyzed by flow cytometry. Data analysis was performed with flowjo software. Statistical analysis was done using Student’s *t*-test for unpaired samples (*p < 0.05; **p < 0.005; ***p < 0.0005).

We first analyzed the frequency of IL-10-producing B cells in the PBMCs of healthy controls and leprosy patients to determine whether *Mycobacterium leprae* infection induces Breg response in leprosy patients. Immunosuppressive IL-10 cytokine is crucial to Breg function; therefore, we checked the expression of IL-10 in CD19^+^ B cells. PBMCs of all three groups were cultured with *M. leprae* sonicated antigen, stained for surface CD19 and intracellular IL-10 and analyzed by flow cytometry. In healthy individuals, very few B cells secreted IL-10 under cultured conditions The frequencies of IL-10^+^ producing B cells significantly increased in lepromatous (BL/LL) patients and tuberculoid (BT) patients as compared to healthy controls (Figures 1A,B). Gating strategy was given as supplementary figure (Figure S1 in Supplementary Material).

Analysis of IL-10 levels in serum samples revealed that lepromatous (BL/LL) patients and tuberculoid (BT) patients produced high levels of IL-10 response in serum as compared to compared to healthy controls (Figure 1C). PBMCs of all three groups stimulated with *M. leprae* sonicated antigen and intracellular IL-10 stained with CD3^+^ T cells and Tregs (CD4^+^CD25^+^FoxP3^+^) cells. We found similar results as of our previous studies demonstrating that T cells and Tregs (CD4^+^CD25^+^FoxP3^+^) cells played a crucial role in immune suppression by secreting IL-10 cytokine (data not shown). Our results collectively show that IL-10 producing Bregs increased from BT to BL/LL pole of leprosy patients and suggesting an immunomodulatory role of these Bregs. These Bregs may be immunosuppressive in nature and may have the capacity to change the plasticity of T cells.

### CD19^+^IL-10^+^ Cells Have Increased Expression of FoxP3 and PDL-1 in Leprosy Patients

**Figure 2:**
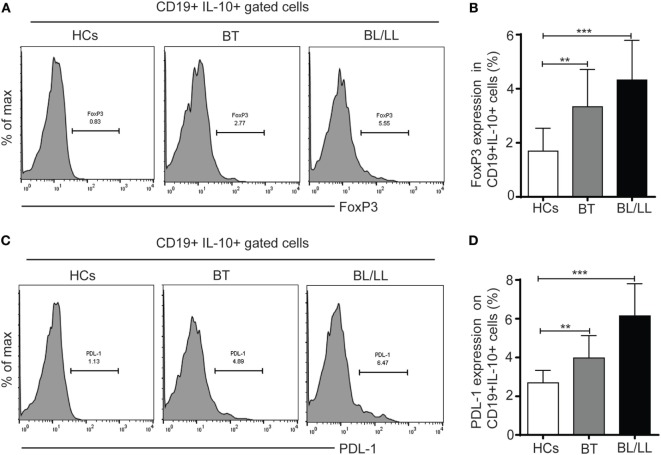
FoxP3 and PDL-1 expression in regulatory B cells (Bregs) (CD19^+^IL-10^+^). **(A)** Representative histogram showing expression of FoxP3 in Breg (CD19^+^IL-10^+^) cells in peripheral blood mononuclear cells (PBMCs) isolated from peripheral blood of HCs (*n* = 10), borderline tuberculoid (BT), and BL/LL patient (*n* = 25). **(B)** Graph plot is showing expression of FoxP3 in HCs and leprosy patients (BT, BL/LL). **(C)** Representative histogram showing expression of inhibitory molecule receptor (PDL-1) on Breg (CD19^+^IL-10^+^) cells in PBMCs isolated from peripheral blood of HCs, BT, and BL/LL patient. **(D)** Graph plot is showing expression of PDL-1 on HCs and leprosy patients (BT, BL/LL). Mean ± SD values are shown in each set while *p* value < 0.05 was considered significant. 100,000 cells were acquired and analyzed by flow cytometry. Data analysis was performed with flowjo software. Statistical analysis was done using Student’s *t*-test for unpaired samples (*p < 0.05; **p < 0.005; ***p < 0.0005).

TGF-β is known to induce FoxP3 expression in Tregs and others immune cells (24) and high TGF-β level has been reported in leprosy patients (4, 25), thus we examined the FoxP3 expression in CD19^+^IL-10^+^ cells derived from leprosy patients and healthy controls. We found 3.33 ± 1.13 and 4.91 ± 1.47% CD19^+^IL-10^+^ cells express FoxP3 in BT and BL/LL patients, respectively, as compared to the healthy control 1.69 ± 0.84% (Figures 2A,B). PD-1-PDL-1 pathway plays important role in the dampening of T cell immune responses during *Mycobacterium* infections. PD-1 is an inhibitory molecule expresses on T cells and its ligands PDL-1 expresses on B cells and other antigen presenting cells. So, we evaluated the expression of PD-1 ligand (PDL-1) on IL-10 producing Bregs by flow cytometry in stimulated PBMC cultures with *M. leprae* antigens. We observed significant higher frequency of PD-L-1 expression on CD19^+^IL-10^+^ cells (Bregs) in BL/LL patients (6.14 ± 1.65, *p* < 0.0005) as compared to healthy controls (2.69 ± 0.64) and BT/TT patients (3.97 ± 1.15, *p* < 0.005) compared to healthy controls (Figures 2C,D). The higher frequency of CD19^+^IL-10^+^ Breg cells with increased expression of FoxP3 and PDL-1 on Bregs suggests that increased in FoxP3 and PDL-1 expression by the Breg cells might be instrumental in dampening of the T cell function in leprosy patients. However, PDL-1 expression on other antigen presenting cells may be one of contributing factors to the immune suppression in leprosy patients.

### CD19^+^IL-10^+^ Cells Converted CD4^+^CD25^−^ Cells Into CD4^+^ CD25^+^ Tregs

**Figure 3:**
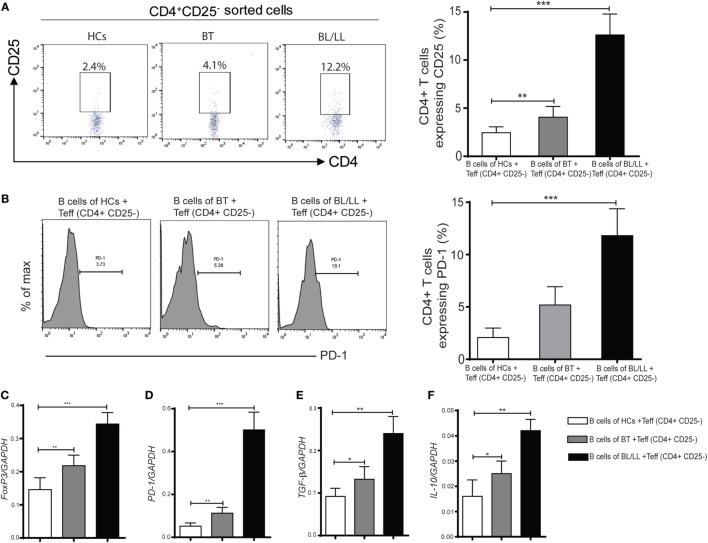
Effect of regulatory B cells (Bregs) on Teff (CD4^+^CD25^−^) cells. **(A)** The percentages of Tregs (CD4^+^CD25^+^) in various groups [HCs, borderline tuberculoid (BT) and BL/LL, *n* = 10] showing in flow diagram. Bregs (CD19^+^IL-10^+^) derived from (HCs, BT, and BL/LL) were cocultured with Teff (CD4^+^CD25^−^) derived from HCs and Tregs were studied by flow cytometry. Graph plot is showing percentage of Tregs in the coculture (Bregs of HCs, BT and BL/LL with Teff of HCs, *n* = 10). **(B)** The percentages of inhibitory molecules (PD-1) on Teff (CD4^+^CD25^−^) in various groups (HCs, BT, and BL/LL) showing in flow diagram. Bregs (CD19^+^IL-10^+^) derived from (HCs, BT, and BL/LL) were cocultured with Teff (CD4^+^CD25^−^) derived from HCs and expression of PD-1 on were studied by flow cytometry. Graph plot is showing percentage of PD-1on Tregs in the coculture (Bregs of HCs, BT, and BL/LL with Teff of HCs). **(C–F)** mRNA expression of FoxP3, PD-1, TGF-β, and IL-10 in the Teff (CD4^+^CD25^−^) of HCs, BT, and BL/LL cocultured with Teff. Mean ± SD values are shown in each set while *p* value < 0.05 was considered significant. 50,000 cells were acquired and analyzed by flow cytometry. Data analysis was performed with flowjo software. Statistical analysis was done using Student’s *t*-test for unpaired samples (*p < 0.05; **p < 0.005; ***p < 0.0005).

It has been reported that Teff (CD4^+^CD25^−^) cells could be converted into Treg under certain circumstances (24, 26, 27). So, we focused on studying the effect of IL-10 producing Bregs on Teff (CD4^+^CD25^−^) cells. We determined the purity of MACS sorted cells, we observed CD19^+^ B cells CD4^+^CD25^−^ cells and CD4^+^CD25^+^ T cells were 98.5%, 98.4% and 97.3% were pure, respectively (Figure S4 in Supplementary Material). To evaluate the effect of CD19^+^ B cells on the plasticity of T effector cells (CD4^+^CD25^−^) cells we cocultured CD19^+^ B cells with CD4^+^CD25^−^ cells (Figure S2 in Supplementary Material). CD19^+^ B cells derived from healthy controls, tuberculoid leprosy patients (BT), and lepromatous leprosy patients (BL/LL) were cocultured with CD4^+^CD25^−^ cells (T effector cells) isolated from healthy controls. We observed that significant number of T effector cells (CD4^+^CD25^−^) were converted into CD4^+^CD25^+^ Tregs when CD4^+^CD25^−^ cells were cocultured with CD19^+^ B cells isolated from BT (4.11 ± 1.12%, *p* < 0.005) and BL/LL leprosy (12.61 ± 2.18%, *p* < 0.0005) patients (Figure 3A). However, healthy controls derived CD19^+^ B cells not significantly able to induce CD25 (2.41 ± 0.86%) expression on CD4^+^CD25^−^ cells. We also evaluated the effect of exogenous IL-10 on CD4^+^CD25^−^ T cells (Figure S5 in Supplementary Material). Next, we also evaluated the PD-1 expression in these CD4^+^CD25^−^ cocultured cells. We found BL/LL derived CD19^+^ B cells induced PD-1 (*p* < 0.0005) expression in CD4^+^CD25^−^ cells, CD19^+^ B cells isolated from BT leprosy (*p* < 0.0005) as compared to healthy controls (Figure 3B). These results were further supported by mRNA expression of FoxP3, PD-1, IL-10, and TGF-β genes expression in cocultured cells. Bregs isolated from BL/LL patients significantly increase the of FoxP3, PD-1, IL-10, and TGF-β expression in Teff cells derived from HCs (Figures 3C–F). Taken together, these results suggested that CD19^+^ B cells of leprosy patients (BT and BL/LL) converted T effector (CD4^+^CD25^−^) cells into Tregs (CD4^+^CD25^+^). Moreover, CD19^+^ B cells of leprosy patients (BL/LL) also induces FoxP3, PD-1 expression as well as also induced IL-10 and TGF-β immunosuppressive cytokines expression in T effector cells. CD19^+^ B cells of leprosy suppress the host immune system by modulating the fate of T effector cells.

### Breg (CD19^+^IL-10^+^) Cells Enhanced Regulatory T-Cells Function in Human Leprosy

**Figure 4:**
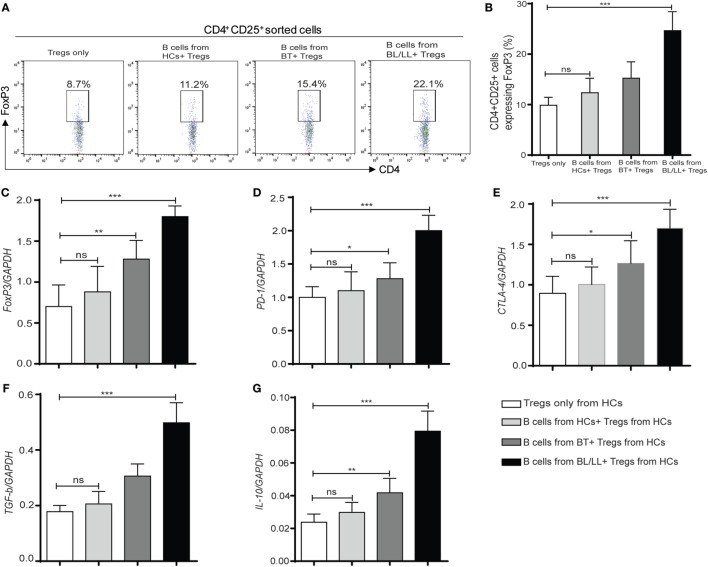
Regulatory effect of regulatory B cells (Bregs) on Tregs (CD4^+^CD25^+^). **(A,B)** Flowcytometric and graph plot representations of CD4^+^CD25^+^ T cells expressing FoxP3. Bregs (CD19^+^IL-10^+^) derived from [HCs, borderline tuberculoid (BT) and BL/LL, *n* = 10] were cocultured with Tregs (CD4^+^CD25^+^) derived from HCs (*n* = 10) and Tregs were studied by flow cytometry. **(C–G)** mRNA expression of FoxP3, inhibitory molecules (PD-1, CTLA-4), TGF-β, and IL-10 in Tregs (CD4^+^CD25^+^) cocultured with Bregs isolated from HCs, BT, and BL/LL (*n* = 10). Mean ± SD values are shown in each set while *p* value < 0.05 was considered significant. 50,000 cells were acquired and analyzed by flow cytometry. Data analysis was performed with flowjo software. Statistical analysis was done using Student’s *t*-test for unpaired samples (*p < 0.05; **p < 0.005; ***p < 0.0005).

CD4^+^CD25^+^ Tregs were known to have a crucial role in the regulation of leprosy disease development and suppression of host immune response according to previous studies (9, 22). So, we wanted to understand the effect of Bregs in leprosy patients on Tregs. With this intention, we cocultured the CD19^+^ B cells isolated from healthy controls, BT, BL/LL patients with CD4^+^CD25^+^ Treg derived from healthy controls and checked changes in Treg functions *in vitro*. Flow cytometry results showed that the expression of FoxP3 in CD4^+^CD25^+^ cells Treg significantly increased (*p* < 0.0005) when leprosy derived (BT, BL/LL) CD19^+^ B cells cocultured with Tregs (CD4^+^CD25^+^) (Figures 4A,B). However, CD19^+^ B cells derived from healthy controls were unable to enhance the FoxP3 expression in Tregs significantly in the coculture. Real-time PCR results revealed that the expression of Treg transcription factor (FoxP3) was also enhanced in the CD19^+^B (BT, BL/LL) + Treg group compared with the healthy control B cells + Treg group (Figure 4C). The expression of inhibitory molecule of T cells and Treg functional molecule PD-1, CTLA-4, and the inhibitory cytokine TGF-β, IL-10 also followed the similar trend as Foxp3 (Figures 4C–E). Collectively, our results showed that anti-inflammatory IL-10 secreted by B cells endowed Treg cells with increased functionality and expression of FoxP3^+^ iTregs with enhanced PD-1 and CTLA-4.

### IL-10 Signaling Blockade Suppresses Regulatory T-Cells Function in Human Leprosy

**Figure 5:**
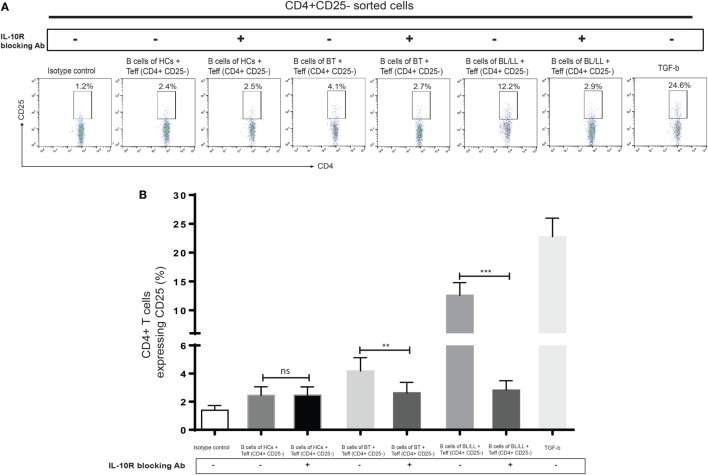
Effect of interleukin-10 (IL-10) blocking signaling of regulatory B cells (Bregs) on Teff. **(A,B)** Representative flow cytometric analysis and graph plot ofTregs (CD4^+^CD25^+^). Bregs isolated from HCs, BT, and BL/LL (*n* = 6) blocked with antibody against IL-10R and cocultured with Teff and expression of CD25^+^ on CD4^+^ was analyzed by flowcytometry after 48 h of culture. Isotype controls and TGF-β (20 ng/ml) were taken as negative and positive controls in both experiments. Mean ± SD values are shown in each set while *p* value < 0.05 was considered significant. 50,000 cells were acquired and analyzed by flow cytometry. Data analysis was performed with flowjo software. Statistical analysis was done using Student’s *t*-test for unpaired samples (*p < 0.05; **p < 0.005; ***p < 0.0005).

**Figure 6:**
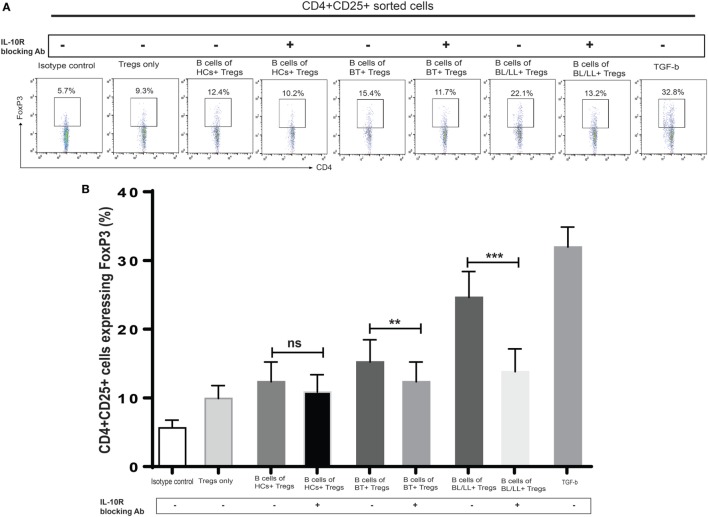
Effect of interleukin-10 (IL-10) blocking signaling of regulatory B cells (Bregs) on Tregs. **(A,B)**. Representative flow cytometric analysis and graph plot of FoxP3 expression in Tregs (CD4^+^CD25^+^). Bregs isolated from HCs, BT, and BL/LL (*n* = 6) blocked with antibody against IL-10 and cocultured with Tregs (CD4^+^CD25^+^) and expression of FoxP3 in CD4^+^CD25^+^ was analyzed by flowcytometry after 48 h of culture. Isotype controls and TGF-β (20 ng/ml) were taken as negative and positive controls in both experiments. Mean ± SD values are shown in each set while *p* value < 0.05 was considered significant. 50,000 cells were acquired and analyzed by flow cytometry. Data analysis was performed with flowjo software. Statistical analysis was done using Student’s *t*-test for unpaired samples (*p < 0.05; **p < 0.005; ***p < 0.0005).

We further tested this hypothesis by taking a pharmacological approach to interrupt IL-10 signaling in Teff and Tregs. Specifically, we used anti-IL-10 receptor monoclonal antibody (aIL-10r mAb) to block IL-10 signaling in Teff and Tregs. We cocultured CD19^+^ cells with effector anti-IL-10 receptor blocked effector T cells and Tregs. We cocultured CD4^+^CD25^−^ and CD4^+^CD25^+^ T cells with isotype antibody as negative control, and TGF-β (20 ng/ml) used as positive control. In this experiment, we observed that Bregs are not able to covert the effector T cells into Tregs (Figures 5A,B) and, also functional activity Tregs is also affected by blocking of IL-10 receptors on Tregs (Figures 6A,B). We also found that the inhibitory molecule (PD-1) and FoxP3 expression in Tregs downregulated by IL-10 receptor blocking (Figure S3 in Supplementary Material). Collectively, the blocking of IL-10R results suggesting that IL-10 producing Bregs facilitates leprosy pathogenesis by inducing the expression of FoxP3 and inhibitory molecule PD-1 and function of T-regulatory cells and, also by converting the effector T cells into Tregs which play important role in immunosuppression of the host immune system toward *M. leprae* thus aiding in the pathogenesis of leprosy.

## Discussion

Results from numerous studies have shown that IL-10 secreted by different cells play an important role for the pathogenies of leprosy (4, 8, 28). It exerts its immunosuppressive function at several levels of the immune system, including modulation of antigen presentation by APCs (29), inhibition of T cell proliferation (30), enhancing and maintaining the function of Tregs (31). The findings of this study encompass the role of IL-10 secreted by Bregs, in addition to TGF-β, IL-10 enhances Tregs differentiation and function. Studies also have shown that increase in TGF-β and IL-10 producing cells are associated with suppression mediated by T-regulatory cells (8).

T-regulatory cells are immunosuppressive cells and exert their immunosuppressive action through the production of IL-10 (28), IL-35 (5), and TGF-β (25). Breg cells also prohibited the expansion T cells and other pro-inflammatory lymphocytes and enhance the function of Tregs (32). In our study, the frequency of Breg cells showed significant increase in the lepromatous pole (BL/LL) of the disease compared to BT and healthy controls. This is in validation with recently published data from leprosy by Negera et al. (33). These Breg cells also produce significant amounts of immunosuppressive IL-10 in leprosy patients BL/LL and BT/TT demonstrating that these cells are suppressive in nature. The transcription factor (FoxP3) was considered as specific intracellular regulatory marker of CD4^+^CD25^+^ T-regulatory cells for several years. Several studies also suggested that FoxP3 is expressed in B cells, macrophages and cancer cells (34, 35). Next, we analyzed the FoxP3 expression in Bregs by flow cytometry and we observed significantly increased expression of FoxP3 in Bregs as we move from BT to BL/LL pole of the disease.

Programmed death-ligand 1 is suppressive molecule, constitutively expresses on B and T lymphocytes, DCs, and monocytes (36, 37). The PD-1/PD-L1 signaling has been shown to be a key player in maintaining peripheral tolerance; however, it is not known whether PD-1/PD-L1 expressed on Bregs acts exclusively on Tregs in leprosy. With this intent we looked for the surface expression of the inhibitory molecule receptors (PD-L1) ligands on B cells, our results showed a high frequency of PD-L1 Breg cells in BL/LL cases as compared to BT. In conjunct to the established role of PD-L1 on Bregs, we hypothesized that Bregs expressing PDL-1 possibly aids in the hasty recruitment of Tregs in leprosy patients. Such heightening of T-regulatory cells may facilitate propagation of disease and suppressed the immune system of the host. This may be one of possible mechanisms utilized by Tregs for immune suppression.

Many cell types like Tregs, macrophages, and DCs etc. secreted IL-10 (12). Our data have indicated that a population of B cells in the leprosy patients constitutively produced IL-10 (Bregs) and this population was greater in abundance in the lepromatous leprosy (BL/LL). IL-10 is one of the cytokine which has key immunomodulatory function on T cells and several studies established the both immunosuppressive and immunostimulatory effects of IL-10 on T cells (38). Therefore, we hypothesized that IL-10 secreted by Bregs can similarly has an effect of T cells function as shown by various studies that T effector could be converted into Tregs under certain circumstances (26, 35). We first showed that CD4^+^CD25^−^ converted into CD4^+^CD25^+^ cells when cocultured with CD19^+^ cells derived from leprosy patients. The real-time PCR assay of FoxP3 and PD-1 further supported the findings that CD19^+^ cells of leprosy patients have capacity to convert the T effector cells into Tregs. Further, we evaluated the effect of Bregs on Tregs, our findings are in agreement with the results of a recent study showing that IL-10 from Bregs is important for the induction and maintenance of Foxp3 expression (31). The percentage of CD4^+^Foxp3^+^ Tregs also enhanced in the presence of leprosy derived Bregs, which indicated that Bregs not only converted the T effector into Tregs itself but also enhanced the Tregs activity. Tregs exhibit immunosuppressive and self-tolerant functions by direct cell contact through membrane expression of programmed death (PD-1), cytotoxic T-lymphocyte associated antigen-4 (CTLA-4) as well as inhibitory cytokines such as interleukin (IL)-10 and transforming growth factor-β (22). The real-time PCR results of FoxP3, PD-1, CTLA-4, TGF-β, and IL-10 further confirmed these findings. IL-10 producing cells function are critical in the immunopathogenesis of many disease states (27, 39). This encouraged us to block the IL-10 secreted cytokines Bregs *in vitro* in the PBMC culture of leprosy patients and see its impact on the effector T cells and Tregs in terms of activity. Blocking of IL-10 cytokine resulted in rescue of effector T cells and diminishing in the function of Tregs. This indicates that by refuting the influence of immunosuppressive IL-10 cytokine we can successfully restore the beneficial effector T cells response and limit the Tregs activity in leprosy patients.

**Figure 7:**
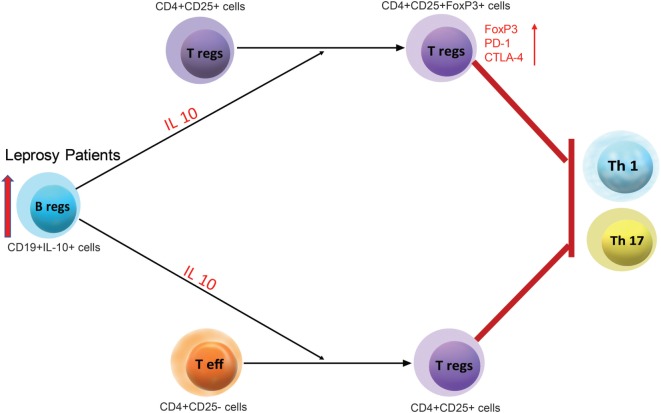
A schematic representation for the regulatory function of leprosy derived regulatory B cells (Bregs) (CD19^+^IL-10^+^) and its regulatory function on Teff (CD4^+^CD25^−^) and Tregs (CD4^+^CD25^+^).

In conclusion, our study evidently demonstrates that IL-10 secreting Bregs are a functionally distinct cell subset that influences the fate of T cells in leprosy patients. These cells convert effector T cells into Tregs and enhance Tregs activity which interns aid in the pathogenesis of leprosy (Figure 7). This study also suggests that further investigation of IL-10 secreting Bregs will assist and help in developing novel Bregs-targeted immunotherapies.

## Ethics Statement

The Institute Ethics Committee, All India Institute of Medical Sciences (AIIMS), New Delhi, India (IESC/T-417/01.11.2013). Written consent was informed obtained from the patients prior to take blood sample.

## Author Contributions

MT, HN, CS, and RAN designed experiments; MT, SK, and CS performed experiments and analyzed the data. MT, HN, RAN, MS, and DNR wrote the manuscript, and NK contributed to clinical diagnosis. AS, RR, and DNR critically reviewed the manuscript. All authors discussed the results and contributed to the final manuscript and reviewed manuscript.

## Conflict of Interest Statement

The authors declare that the research was conducted in the absence of any commercial or financial relationships that could be construed as a potential conflict of interest.
