# Supplementary figures and images for: Interleukin-10 Producing Regulatory B Cells Transformed CD4+CD25− Into Tregs and Enhanced Regulatory T Cells Function in Human Leprosy
Source: Front Immunol. 2018 Jul 23;9:1636. doi: 10.3389/fimmu.2018.01636 (PMC6065098; doi:10.3389/fimmu.2018.01636)

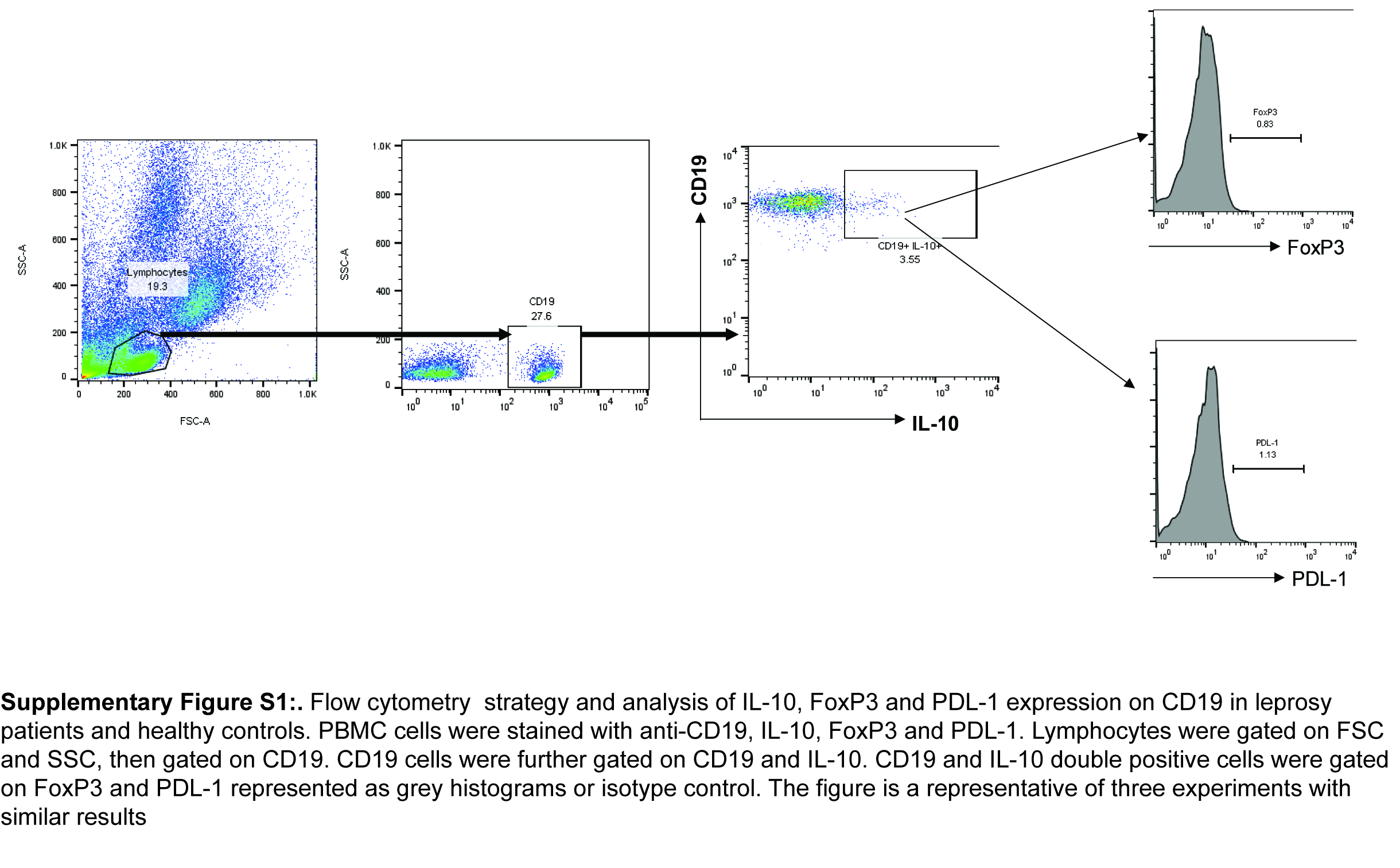

Supplement: Supplementary file 1 [file image_1.tif]

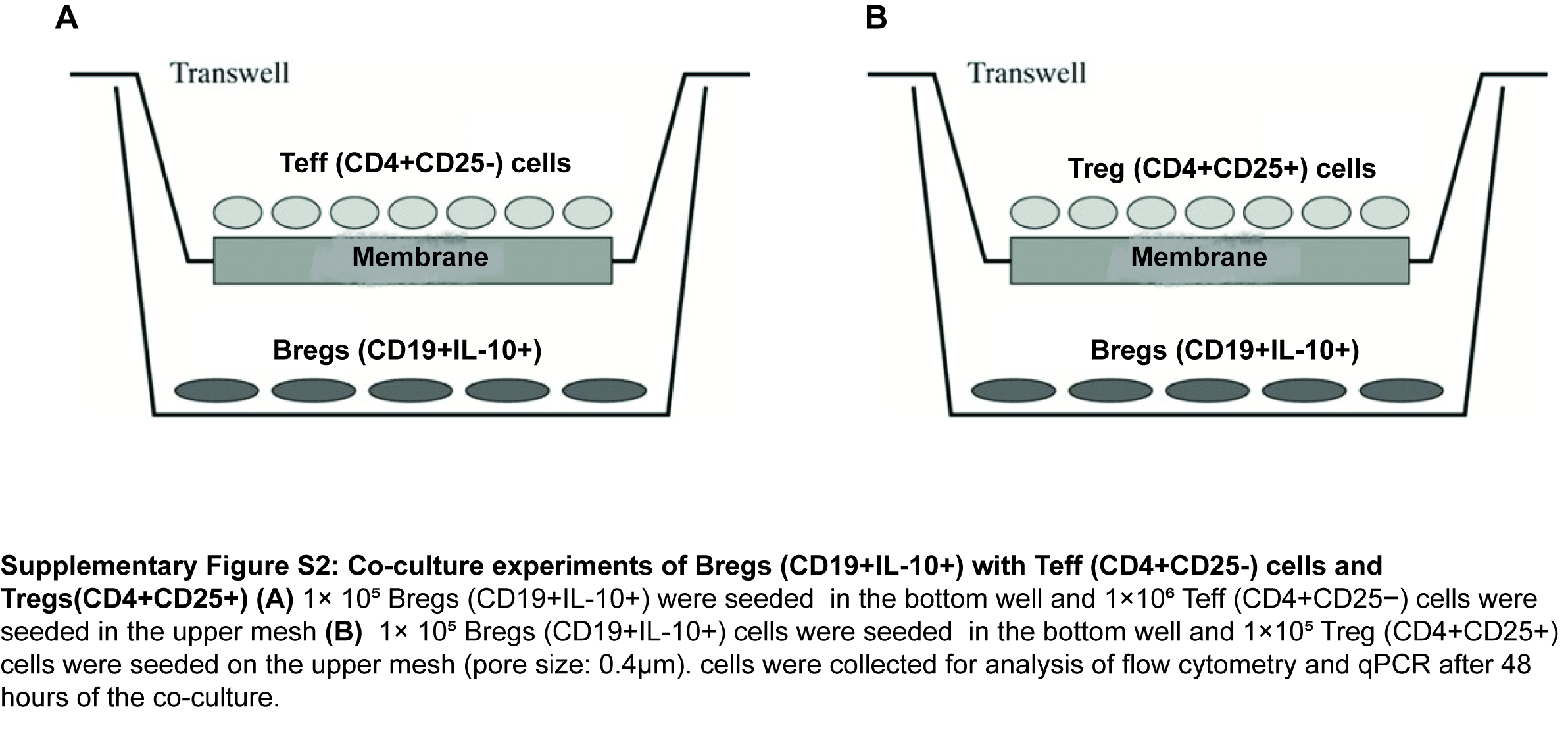

Supplement: Supplementary file 2 [file image_2.tif]

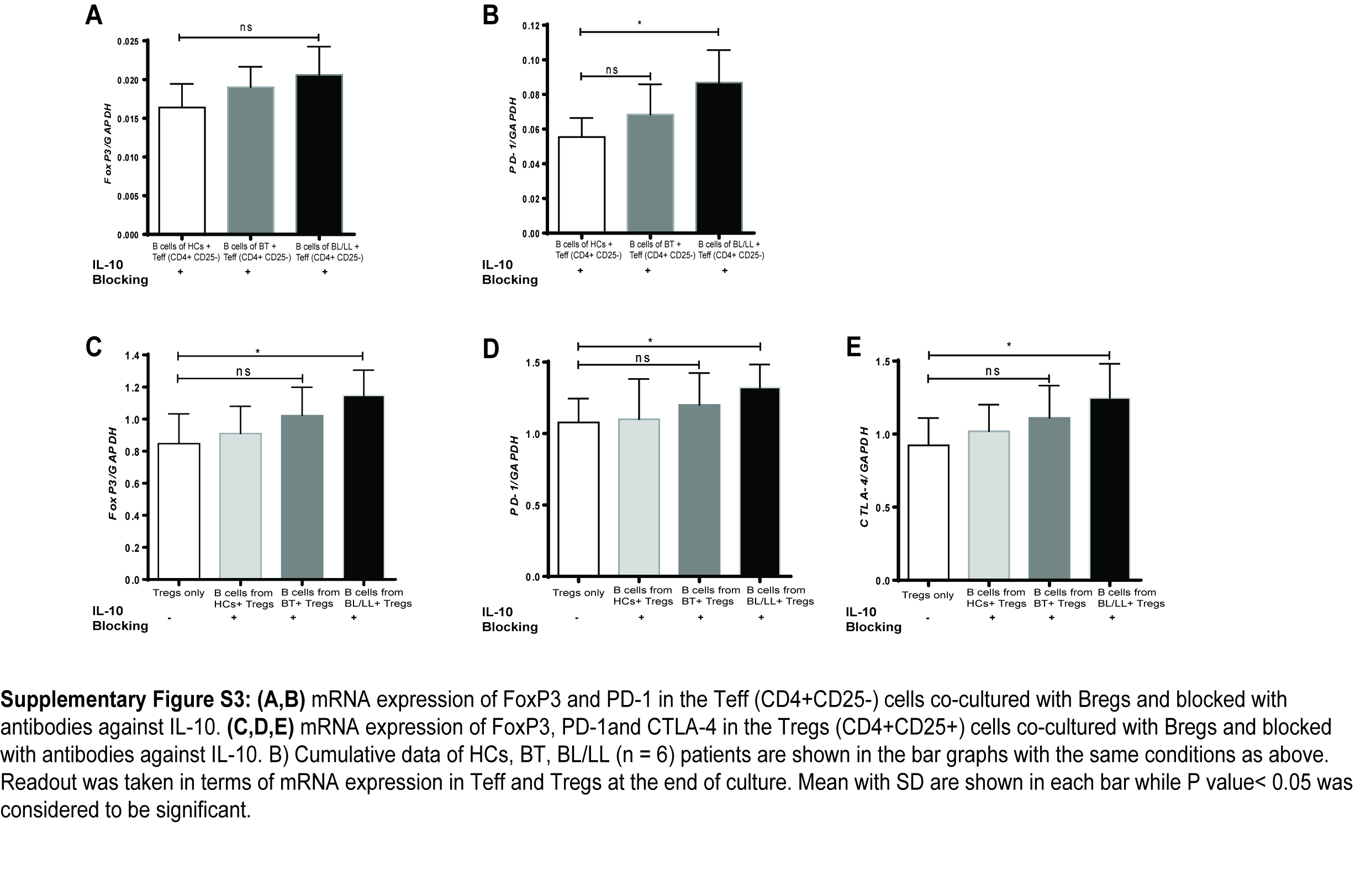

Supplement: Supplementary file 3 [file image_3.tif]

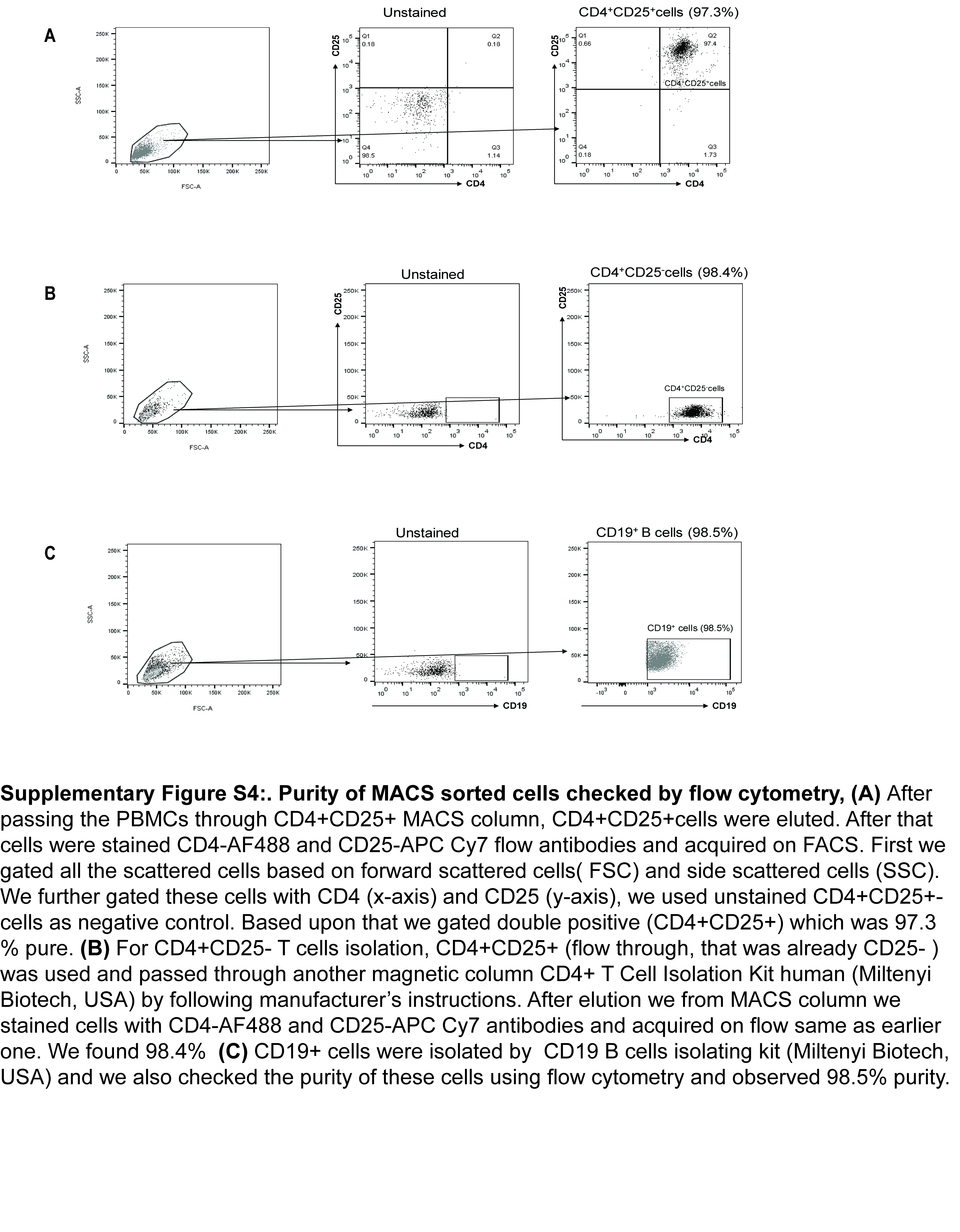

Supplement: Supplementary file 4 [file image_4.tif]

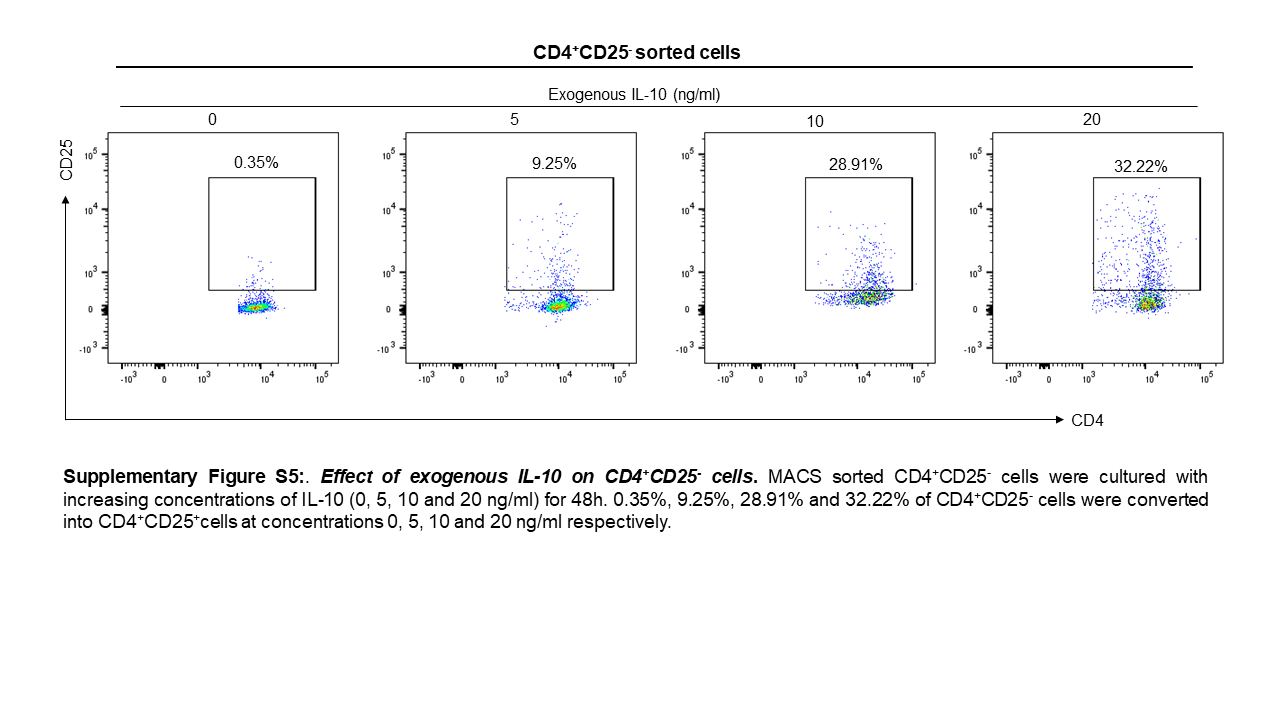

Supplement: Supplementary file 5 [file image_5.tif]
